# EBV‐associated lymphoproliferative disorder in a patient with X‐linked severe combined immunodeficiency with multiple reversions of an *IL2RG* mutation in T cells

**DOI:** 10.1002/jha2.119

**Published:** 2020-10-30

**Authors:** Fumiya Wada, Tadakazu Kondo, Momoko Nakamura, Shunsuke Uno, Masakazu Fujimoto, Takayuki Miyamoto, Yoshitaka Honda, Hirofumi Shibata, Kazushi Izawa, Takahiro Yasumi, Momoko Nishikori, Akifumi Takaori‐Kondo

**Affiliations:** ^1^ Department of Hematology and Oncology Graduate School of Medicine Kyoto University Kyoto Japan; ^2^ Department of Diagnostic Pathology Graduate School of Medicine Kyoto University Kyoto Japan; ^3^ Department of Pediatrics Graduate School of Medicine Kyoto University Kyoto Japan

To the Editor,

Kawai et al reported the case of a patient with X‐linked severe combined immunodeficiency (XSCID) having multiple reversions of an *IL2RG* mutation in T cells. XSCID is caused by mutations in the gene coding for common gamma chain (γc) of interleukin‐2 receptor (*IL2RG*), characterized by the lack of T cells and NK cells, and shows hypogammaglobulinemia despite normal B‐cell count [1]. In the XSCID patients, persistent infections with the opportunistic organisms uniformly lead to death within the first 2 years of life without allogeneic hematopoietic stem cell transplantation (allo‐HSCT), except for those with atypically attenuated phenotypes. This patient is among the six XSCID patients with genetic reversions reported so far [1, 2, 3, 4, 5, 6], in which both the number of CD4+ and CD8+ T cells and T‐cell proliferation capacity had been maintained because of multiple somatic reversions of an *IL2RG* mutation in T cells. Although the patient had a milder clinical course with immunoglobulin replacement therapy for at least 20 years due to genetic reversions, questions remained on the longevity of the revertant cells with protective immune function. He consequently developed Epstein‐Barr virus associated lymphoproliferative disorder (EBV‐LPD) at 21 years of age. Here, we report the first case of EBV‐LPD followed by SCID with reversions of an *IL2RG* mutation in T cells.

Laboratory investigations conducted at the of 21 years revealed liver dysfunction. Abdominal ultrasonography found multiple paraaortic lymphadenopathies; however, lymph node biopsy by laparoscopy could not confirm the diagnosis. About 6 months later, he developed pancytopenia (WBC: 950/μL, Hb: 10.3 g/dL, and PLT: 24 × 10^3^/μL). Although bone marrow examination did not show any specific findings, ^18^F fluorodeoxyglucose‐positron emission tomography/computed tomography (^18^F‐FDG‐PET/CT) demonstrated abnormal uptake in lymph nodes, spleen, bone marrow, and upper pharynx (Figure S1A). Therefore, we planned a re‐biopsy, but it could not be performed because of persistent high fever and severe thrombocytopenia. High fever nonresponsive to antibiotics and increased levels of serum ferritin and lactate dehydrogenase (LDH) were suggestive of hemophagocytic lymphohistiocytosis (HLH). After starting prednisolone (1 mg/kg), recurrent fever and pancytopenia were improved. A diagnostic biopsy of the intraabdominal lymph node revealed large scattered tumor cells positive for CD30, IMP3, EBER, and CD15 (partial), but negative for B‐ and T‐cell markers. A diagnosis of Hodgkin lymphoma (HL)‐like EBV‐LPD (Ann Arbor stage IV) was made based on these findings. High load of EBV‐DNA was found in the peripheral blood sample (650 copies /μg DNA). The proportions of the lymphocyte subsets of CD4+ T cells, CD8+ T cells, CD19+ B cells, and CD56+ NK cells in the peripheral blood were 31.9%, 60.2%, 2.3%, and 0.3%, respectively (total lymphocyte count was 260/μL). Expression of common gamma chain (also known as CD132) at the time of diagnosis of LPD was compared with the previous level at the age of 9 years (Figure 1A). Expression level of CD132 was maintained in T cells but reduced in B cells and monocytes, indicating that T‐cell function was comparable to that in his childhood. However, immunophenotypic analysis of T cells demonstrated remarkably decreased number of naïve CD4+ T cells (CD4+CD45RA+CD27+) (Table 1).

**FIGURE 1:**
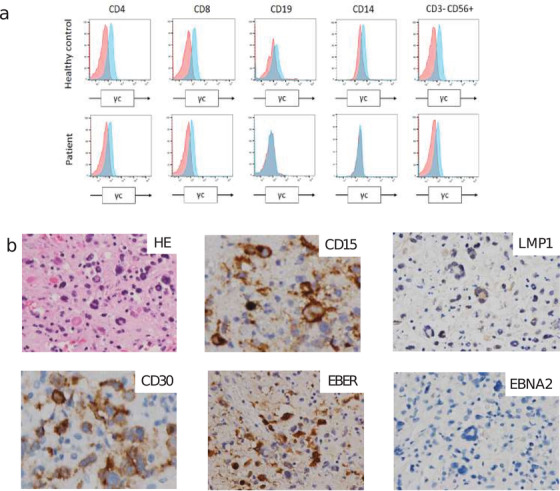
(A) Surface expression of the common γ‐chain on the peripheral blood mononuclear cells (PBMCs) from the patient and healthy control gated according to the expression of the indicated lineage surface markers at 9 years of age. Blue lines indicate staining for the common γ‐chain (with anti‐CD132 Ab) and red lines indicate staining with the isotype control. (B) Histology of the lymph node (HE; original magnification × 600, CD15; × 600, CD30; × 600, EBER; × 600, LMP1; × 600, and EBNA2; × 600)

**TABLE 1:** Flow cytometric analysis of T cells at the time of EBV‐LPD diagnosis, compared to an age‐matched control

	Patient (%)	Healthy control (%)
CD4+/lymphocyte	19.4	37.6
Naïve CD4+ T‐cell ratio (CD4+CD45RA+CD27+/CD4+)	5.2	62.1
CD8+/lymphocyte	63.5	42.7
Naïve CD8+ T‐cell ratio (CD8+CD45RA+CD27+/CD8+)	23.9	30.5
Memory CD8+ T cell ratio (CD8+CD45RA−/CD8+)	18.2	7.8
Effector CD8+ T cell ratio (CD8+CD45RA+CD27−/CD8+)	58.0	61.8

After the diagnosis of EBV‐LPD, treatment with brentuximab vedotin (BV) (1.8 mg/kg every 3 weeks) was initiated, owing to presumed intolerance to multiagent chemotherapy due to immunodeficiency and pancytopenia. Follow‐up CT study revealed decreased hepatosplenomegaly and abdominal lymphadenopathy after the first cycle of BV therapy. However, after five cycles, high fever of around 40°C recurred and liver dysfunction and pancytopenia were indicated by laboratory testing. Therefore, we started antibiotics treatment, although CT scans and blood culture revealed no signs of infection. Bone marrow smears suggested the invasion of tumor cells and ^18^F‐FDG‐PET showed abnormal uptakes in the bone marrow, while FDG uptake was decreased in the abdominal lymph nodes and hepatomegaly. According to these findings, we intended to administer gemcitabine to control LPD refractory to BV. However, he suddenly developed septic shock and disseminated intravascular coagulation, followed by rapid deterioration of the condition and death within a day after sudden change. *Corynebacterium minutissimum* and *Staphylococcus caprae* were detected in the blood culture.

Autopsy was performed to determine the extent of tumor involvement, followed by comparison of the pathological diagnosis with the clinical diagnosis. Unexpectedly, LPD was disseminated to whole body including the porta hepatis, lymph node, liver, spleen, lungs, bone marrow, and adrenal gland. Histology of the lymph node showed tumor cells with large nuclei that were positive for CD15, CD30, and EBER, partially positive for PD‐L1, and negative for B, T, and NK cell markers (Figure 1B). In addition, tumor cells were positive for LMP1, but negative for EBNA2, suggesting that EBV latency showed a latency II pattern (Figure 1B). Southern blot analysis of EBV‐DNA confirmed clonal proliferation of EBV‐positive cells in the spleen (Figure S1B). Southern blot analysis of T‐cell receptor (TCR) *β* and *γ* genes demonstrated clonal rearrangement in the *γ* but not in the *β* gene (Figure S1C). In addition, multiplex PCR analysis of both *TCR β* and *γ* genes suggested the presence of an oligoclonal T‐cell population. Since originally he was considered to have limited TCR diversity, we assumed that these results did not indicate T‐cell malignancy.

The development of EBV‐LPD in the individuals with primary immunodeficiency (PID) disease highlights several critical immune pathways that are needed for protection from viral infection and virus‐induced oncogenicity. Among them, the cytotoxic pathways of CTLs and NK cells are crucial for protection from most viral infections and malignancies [7]. Defects in the T‐cell line—ranging from impaired TCR rearrangement and thymic egress to poor long‐term maintenance of cellular immunity and reduced cell activation—affect various steps in T‐cell development and activation [8]. In this case, although reversion mutations in T cells were maintained, remarkably decreased number of naïve CD4+ T cells suggested restricted TCR repertoire. This insufficient T‐cell functions and defect of NK cells might induce EBV‐LPD over a long period. In addition, the number of T cells gradually decreased over a period of time, indicating that T‐cell exhaustion was associated with the development of EBV‐LPD (Table S1). Moreover, there is a possibility that the clonal change was related to the occurrence of EBV‐LPD, although the sequential data of reversion/wild ratios were not obtained due to the nonavailability of samples.

LPD is a recognized complication of PID, with historically very poor outcome. Reported mortality rates of PID associated with LPD is nearing 70% when the disease is unresponsive to conventional chemotherapy [9]. For patients surviving LPD, stem cell transplantation (SCT) is the only cure for the underlying PID [10]. After initial treatment of BV, we considered performing an allo‐SCT to cure LPD and underlying XSCID; however, the SCT could not be performed because of his poor condition. It might be crucial to determine an appropriate time for transplantation to cure LPD‐associated PID even though immunity is appeared to be maintained by the reversion of the mutation.

## AUTHOR CONTRIBUTIONS

FW and TK wrote and edited the manuscript. SU and MF provided the pathology images, wrote the pathology findings, and revised and edited the manuscript. MN, TM, YH, HS, KI, TY, and ATK revised and edited the manuscript. All the authors have read final version of the manuscript and have approved this submission.

## CONFLICT OF INTEREST

The authors declare that they have no conflict of interest.

## Supporting information

Table S1. Change in number of lymphocytes over time.

Figure S1. (a) ^18^F‐FDG‐PET/CT images at the onset of EBV‐LPD. (b) Southern blot analysis of the spleen for EBV‐terminal repeats at autopsy. (c) Southern blot analysis of *T cell receptor (TCR) γ* gene rearrangement. The arrows indicate monoclonal bands. E: *EcoRI* digestion*, B: BanH‐*digestion*, K: Kpn I* digestion

## Data Availability

Data sharing is not applicable to this article as no new data were created or analyzed in this study.
